# Molecular Mechanisms of Ferroptosis and Its Role in Pulmonary Disease

**DOI:** 10.1155/2020/9547127

**Published:** 2020-06-26

**Authors:** Ningning Tao, Kang Li, Jingjing Liu

**Affiliations:** ^1^Department of Respiratory Medicine and Critical Care, Beijing Hospital, National Center of Gerontology, Institute of Geriatric Medicine, Chinese Academy of Medical Sciences, China; ^2^Graduate School of Peking Union Medical College, Beijing 100730, China; ^3^The MOH Key Laboratory of Geriatrics, Beijing Hospital, National Center of Gerontology, Beijing, China

## Abstract

Ferroptosis is a new mode of cell death that is characterized by the excessive accumulation of iron and lipid peroxides. It has unique morphological changes and disparate biochemical features and plays an intricate role in many pathophysiological processes. A great deal of researches confirms that ferroptosis can be regulated by numerous molecules through different mechanisms, supporting great potentials for novel pharmacological therapeutics. Recently, several studies reveal that ferroptosis is also closely associated with the initiation and development of respiratory disease. Understanding the specific mechanism, the molecular trait of ferroptosis and their relationship with pulmonary disease could provide significant references regarding effective treatment of these obstinate disease.

## 1. Introduction

Ferroptosis is a new form of regulated cell death (RCD) that results from the overaccumulation of iron-dependent reactive oxygen species (ROS) and lipid peroxides and is characterized by enhanced mitochondrial membrane density and cell volume shrinkage, differing from other RCDs morphologically, biochemically, and genetically [1].

**Figure 1:**
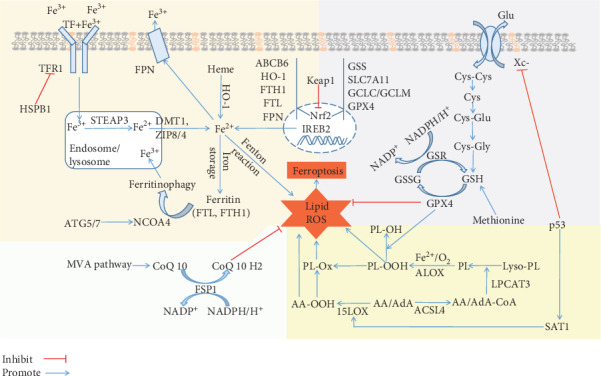
The regulatory mechanisms of ferroptosis: (1) iron metabolism mechanism, including HSPB1-TFR1, ATG5/7-NCOA4 pathway, IREB2 pathway, and Keap1-Nrf2 pathway; (2) system Xc-, including Xc-/GSH/GPX4, p53/SLC7A11 pathway, Keap1-Nrf2 pathway, and sulfur transfer pathway (methionine); (3) lipid metabolism mechanism, including p53-SAT1-15LOX pathway, ACSL4, and LPCAT3; (4) MVA pathway and FSP1-CoQ10-NAD(P)H pathway working cooperatively with GPX4 and GSH/GSSG to inhibit phospholipid peroxidation and ferroptosis. Abbreviations—ACSL4: acyl-CoA synthetase long-chain family member 4; ALOX: arachidonate lipoxygenase; AA: arachidonoyl; AdA: adrenoyl; ABCB6: ATP-binding cassette subfamily B member 6; ATG5: autophagy-related 5; ATG7: autophagy-related 7; CoQ10: coenzyme Q10; Cys: cysteine; system Xc-: cysteine/glutamate transporter receptor; DMT1: divalent metal transporter 1; FTH1: ferritin heavy chain 1; FTL: ferritin light chain; FPN: ferroportin; FSP1: ferroptosis suppressor protein 1; Glu: glutamate; GCLC/GCLM: glutamate-cysteine ligase; GSH: glutathione; GSR: glutathione-disulfide reductase; GPX4: glutathione peroxidase 4; GSS: glutathione synthetase; Gly: glycine; HSPB1: heat shock protein beta-1; HO-1: heme oxygenase-1; IREB2: iron-responsive element binding protein 2; Keap1: Kelch-like ECH-associated protein 1; LOX: lipoxygenase; LPCAT3: lysophosphatidylcholine acyltransferase 3; MVA: mevalonate; NADPH: nicotinamide adenine dinucleotide phosphate; Nrf2: nuclear factor erythroid 2-related factor 2; NCOA4: nuclear receptor coactivator 4; GSSG: oxidized glutathione; PL: phospholipid; ROS: reactive oxygen species; STEAP3: six transmembrane epithelial antigen of the prostate 3; SLC7A11: solute carrier family 7 member 11; SAT1: spermidine/spermine N^1^-acetyltransferase 1; TF: transferrin; TFR1: transferrin receptor 1; ZIP8/14: zinc-iron regulatory protein family 8/14.

Since the concept of ferroptosis was established in 2012 [1], endless efforts have revealed its underlying mechanisms [2]. Up to now, several ferroptosis-related signaling pathways and small molecules have been identified (Figure 1, Table 1). The accumulation of excessive intracellular iron, the depletion of glutathione (GSH), the inactivation of glutathione peroxidase 4 (GPX4), and the upregulation of lipid peroxidation are essential to the initiation and development of ferroptosis [3–6]. Multiple genes, proteins, and cell organelles also have been found to participate in the regulation of ferroptosis [1, 7–10]. These findings support the potential of effective therapies targeting positive and negative modulation of ferroptosis through pharmacologic or genetic intervention.

Emerging evidences have confirmed the contribution of ferroptosis in various physiological and pathological processes, such as development [11], neurodegeneration disease [12, 13], and cancer [14, 15]. In 2017, Wenzel et al. detailed the significance of phosphatidylethanolamine-binding protein 1- (PEBP1-) involved regulatory pathway of ferroptosis in airway epithelial cells in asthma [16]. Moreover, another research finds that the suppression of the iron-sulfur cluster biosynthetic enzyme NFS1 can cooperate with cysteine transport blockage to suppress lung tumor by inducing ferroptosis [17]. The role of ferroptosis in pulmonary disease has become the focus and hotspot of study on exploring novel diagnostic and therapeutic methods for associated disease.

This review focuses on the mechanism, the signaling pathway, the small molecule, and the regulator involved in ferroptotic process and tries to provide a general overview on the relationship between ferroptosis and pulmonary disease.

## 2. Main Text

### 2.1. Ferroptosis: A New Form of RCD

Ferroptosis driven by iron-dependent lipid peroxidation is a new kind of RCD [1]. It is triggered when the deranged intracellular iron homeostasis results in overaccumulation of toxic lipid ROS, exceeding the cell's antioxidation capacity, damaging the membrane structure [18, 19]. The imbalanced oxidation stress and antioxidant status can induce lipid peroxidation and cause lethal damage to lipids, proteins, and nucleic acids [20], and subsequently, it leads to ferroptotic cell death with a condensed mitochondrial membrane, shrunken cell volume, and mitochondria crista that differ from apoptosis and necrosis morphologically [1]. Biochemically, ferroptosis can be suppressed by various small molecules, involving iron chelators (e.g., deferoxamine), lipophilic antioxidants (e.g., *α*-tocopherol), and lipid peroxidation inhibitors [21–24] that to the maximum extent do not similar with other forms of RCD [1, 5, 25, 26]. Moreover, while numerous genes including *p53* [7, 27, 28], nuclear factor E2-related factor 2 (*Nrf2*) [29, 30], and autophagy-related (*ATG*) genes [9, 31] modulate different RCDs, a distinct set of genes such as ribosomal protein L8 (*RPL8*), ATP synthase F0 complex subunit C3 (*ATP5G3*), tetratricopeptide repeat domain 35 (*TTC35*), citrate synthase, and iron response element binding protein 2 (*IREB2*) seems peculiar to regulate ferroptosis [1]. As ferroptosis shares different mechanisms with other forms of cell death, it could unquestionably circumvent their limitations.

### 2.2. The Mechanisms of Ferroptosis

Since the discovery of ferroptosis, increasing evidences have elaborated that it could be triggered and modulated by diverse mechanisms including iron, ROS, and lipid metabolism pathways. Here, the following part comprehensively summarizes the basic mechanisms and key regulators in the ferroptotic process (Figure 1).

#### 2.2.1. Iron and Ferroptosis

As an essential trace element in the human body, iron plays a vital role in survival and biological processes through enzymatic or nonenzymatic reactions [32]. However, too heavy loading of iron can promote the generation of ROS via Fenton reaction and iron-incorporating proteins, facilitating the initiation of ferroptosis [3, 33, 34]. Normally, the intracellular iron was kept in a relative equilibrium through absorption and metabolism. Dietary iron can mainly be absorbed by intestinal epithelial cells in the form of ferric iron; then it binds to circulating glycoprotein transferrin (TF) and is imported into cells by the membrane protein transferrin receptor (TFR1). Intracellular ferrous iron that is transformed from ferric iron through the six transmembrane epithelial antigen of the prostate 3 (STEAP3) in endosomes is stored in a labile iron pool (LIP) in the cytoplasm via zinc-iron regulatory protein family 8/14 (ZIP8/14) or divalent metal transporter 1 (DMT1) [35]. The intracellular iron can be exported out of the cell by ferroportin (FPN, the unique cellular iron exporter) or stored in cell as ferritin. Ferritin can be degraded into ferric iron via the nuclear receptor coactivator 4- (NCOA4-) mediated ferritinophagy pathway [36]. The proteins involved in the aforementioned iron metabolism process (e.g., iron uptake, export, storage, and utilization) and a series of genes targeted to the regulation of iron (e.g., *TFRC*, *IREB2*, *FTL*, *FTH1*, and *FBXL5*) can mediate ferroptosis by modulating the level of intracellular iron [19, 37]. For example, the knocking down of *Fpn* accelerates erastin-induced ferroptosis via reducing iron export [38]. In contrast, the suppression of iron-responsive element-binding protein 2 (IREB2) which encodes the key regulator of iron metabolism obviously alleviates erastin-induced ferroptosis [1]. The increasing of NCOA4-mediated ferritinophagy can promote ferroptosis by enhancing ferritin degradation [39, 40]. Heat shock protein family B member 1 (HSPB1) reduces ferroptosis by inhibiting the TFR1-mediated iron uptake [41]. Iron regulatory protein 2 (IRP2), heme oxygenase 1 (HO-1), and mitochondrial iron exporter CISD1 (CDGSH iron sulfur domain 1) mediate ferroptosis by controlling the level of intracellular free iron [1, 42, 43].

#### 2.2.2. ROS and Ferroptosis

ROS are a class of partially reduced oxygen-containing molecules that include peroxides (H_2_O_2_, ROOH), superoxide anions (O_2_^·-^), and free radicals (R^·^, RO^·^, HO^·^, HO_2_^·^, NO, and NO_2_), which are crucial for the maintenance of cell and tissue homeostasis [44]. The majority of ferroptosis-related ROS are derived from Fenton and Haber-Weiss reactions in which the cellular divalent iron and H_2_O_2_ are catalyzed and decomposed into ferric iron and hydroxyl radicals [45]. It also can be generated from various iron and iron derivatives which incorporated enzyme including nicotinamide adenine dinucleotide phosphate (NADPH) oxidases (NOX), xanthine oxidase (XO), cyclooxygenase (COX), nitric oxide synthase (NOS), lipoxygenases (LOXs), and cytochrome P450 as well as from the mitochondrial electron transport chain [46]. The excrescent ROS are detoxified by enzymatic (e.g., glutathione peroxidase (GPX) [47], superoxide dismutase (SOD) [48], thioredoxin (Trx) [49], and catalase (CAT) [50]) and nonenzymatic antioxidants (vitamins [51], coenzyme Q10 (CoQ10) [52], zinc [53], and melatonin [54]). The excessive generation and insufficient elimination of ROS can lead to oxidative stress and increase the sensitivity of ferroptosis [46]. Both cytosolic ROS eliminator (N-acetylcysteine) and mitochondrial targeted antioxidant (MitoQ) can alleviate erastin and its analogue-induced ferroptosis via blocking ROS production [55]. As a vital intracellular reductant, NADPH can reduce the sensitivity of ferroptosis by eliminating lipid hydroperoxides especially in the central nervous system [1, 56].

#### 2.2.3. Lipid Peroxidation and Ferroptosis

Lipids carry out various functions, from biomembrane composition to energy storage and signaling transmission [6]. Lipid metabolism is essential to the modulation of cellular sensitivity to ferroptosis. The initiation of lipid peroxidation is characterized by the oxidization of lipid species (L), especially the oxidization of polyunsaturated fatty acids (PUFAs) which transfer hydrogen to ROS, reactive nitrogen species (RNS), and reactive lipid species (RLS) and generate a lipid radical (L^·^). The L^·^ and oxygen interact with each other to produce a lipid peroxyl radical (LOO^·^), which can then obtain another hydrogen atom to generate another L^·^ and lipid peroxide (LOOH), contributing to the stage of propagation reaction. The propagation reaction carries on until the radical species are decomposed by the antioxidants or sufficient to yield a stable nonradical product; thus, the chain reaction is terminated [57]. As a substrate of lipid signaling mediators, free PUFAs should be oxidated and esterified into membrane phospholipids before transmitting the ferroptosis signals [58]. Differing from saturated and monounsaturated fatty acids (MUFAs), PUFAs which are extremely sensitive to oxidative stress seem to be essential for ferroptosis [59]. It is reported that PUFA-PEs (phosphatidylethanolamines) which harbor arachidonoyl (AA) and adrenoyl moieties (AdA) are the preferential substrates of oxidation in ferroptosis [58]. The long-chain AA/AdA can be ligated with coenzyme A by Acyl-CoA synthetase long-chain family member 4 (ACSL4), subsequently, AA/AdA-CoA can be esterified into phosphatidylethanolamines catalyzed by lysophosphatidylcholine acyltransferase-3 (LPCAT3), and then the products can be oxidized into lipid hydroperoxides by arachidonate lipoxygenase (ALOX) and finally induce ferroptosis [23]. Once the phospholipid hydroperoxides can not be degraded by GPX4 timely, the excessive lipid peroxides will lead to ferroptosis [58]. The silencing of *ACSL4* and *LPCAT3* which are vital to the membrane phospholipid insertion and remodeling of PE can alleviate ferroptosis via ablating lipid peroxides [23, 60, 61]. The lipoxygenases (LOXs), a nonheme, iron-containing protein, can further oxidate PUFA-PE to facilitate ferroptosis [58]; moreover, the inhibition of LOX by baicalein and nordihydroguaiaretic acid (NDGA) can alleviate ferroptosis [62, 63].

### 2.3. The Signaling Pathways of Ferroptosis

#### 2.3.1. System Xc-

Glutamate-cystine antiport system Xc- composed of a catalytic subunit solute carrier family 7 member 11 (SLC7A11) and a regulatory subunit solute carrier family 3 member 2 (SLC3A2) plays an important role in regulating ferroptosis [1]. The exchange of cystine (import into the intracellular) and glutamate (export into the extracellular space) is mediated by system Xc- in a 1 : 1 ratio [1]. The intracellular cystine can be converted into cysteine which is crucial for GSH synthesis [64]. Moreover, methionine can enhance GSH synthesization in a sulfur-transfer pathway [65]. GSH is vital to protect cells from oxidative damage by reducing ROS and RNS. Buthionine sulfoximine and cisplatin can induce ferroptosis by depleting GSH [5, 66]. While increasing the cystine uptake by using *β*-mercaptoethanol as well as promoting the *SLC7A11* gene transcription by targeting activating transcription factor 4 (*ATF4*) can inhibit ferroptotic process [1, 67], blocking system Xc- by its inhibitors including erastin, sulfasalazine, sorafenib, and glutamate can result in the insufficient cystine absorption, the GSH depletion, the antioxidant capacity reduction, the lipid ROS accumulation, and ferroptosis [22, 68].

#### 2.3.2. GPX4

As an antioxidant enzyme, GPX4 is a robust regulator to prevent ferroptosis by combating lipid peroxidation. Two molecules of GSH can be oxidized into glutathione (GSSG) by selenoenzyme GPX4 and donate electrons to reduce toxic phospholipid hydroperoxides (PL-OOH) into nontoxic phospholipid alcohols (PL-OH) [47]. The GSSG can be reduced by NADPH/H^+^ and glutathione reductase (GSR) to recycle reduced GSH [69]. The inactivation of GPX4 can result in ferroptosis even if the cell is under normal intracellular cysteine and GSH levels, making it different from the system Xc- inhibition which works through inducing GSH depletion [5]. The overexpression of GPX4 can abate the production of ROS; on the contrary, blocking GPX4 by RSL3 and FIN56 can result in ROS accumulation and accordingly promote ferroptosis [5]. In addition to causing an iron- and ROS-dependent ferroptotic process [5, 70], the knockout of *Gpx4* also can damage tissue in a necrotic, apoptotic, or pyroptosis manner [71–73]. Although GPX4 is definite essential to the regulation of ferroptosis, the role of GPX4 in different RCDs, the function of other GPXs, the relationship between GPXs, and other antioxidant pathways are complicated and insufficiently explored.

#### 2.3.3. P53

P53 is well known as a tumor suppressor participating in cell cycle inhibition, apoptosis, and senescence; however, its complicated role in regulating ferroptosis is recently revealed [28, 74]. It is not until 2015 that p53 was deemed as a novel regulator in the ferroptotic process [75]. Subsequently, continuous researches uncover the dual effects of p53 on ferroptosis through either a transcriptional or posttranslational mechanism. On the one hand, p53 is a transcriptional repressor of *SLC7A11* and can facilitate cells to ferroptosis by inhibiting cysteine uptake [75]. P53 can also sensitize ferroptosis by enhancing the expression of *GLS2* (glutaminase 2) and *SAT1* (spermidine/spermine N^1^-acetyltransferase 1). GLS2 participates in the regulation of ferroptosis by decreasing glutathione and increasing cellular ROS levels [76]. SAT1 can promote the expression and activation of 15-LOX, an iron-binding enzyme that enhances PUFA oxidization and lipid peroxidation [77]. On the other hand, p53 can inhibit ferroptosis by restraining the activity of DPP4 (dipeptidyl peptidase 4) directly or by promoting the expression of CDKN1A/p21 (cyclin-dependent kinase inhibitor 1A). In the absence of p53, DPP4 and NOX1 interact to form a complex, which then increases lipid peroxidation and ferroptosis [78]. The p53/p21 pathway can suppress the initiation of ferroptosis, thus protecting cancer cells to survive under the condition of insufficient cystine [79].

#### 2.3.4. Nrf2

Nrf2 is a pivotal transcription factor of the cellular antioxidant response, and its targets play an important role in iron and lipid metabolism [80, 81]. The intracellular Nrf2 homeostasis is modulated by the ubiquitination of a combination that mainly consists of Kelch-like ECH-associated protein 1 (KEAP1) and Cul3 E3 ubiquitin ligase. Under oxidative stress, intracellular Nrf2 and KEAP1 are dissociated from each other; then, Nrf2 is translocated into the nucleus and initiates the transcription of antioxidant and ferroptosis related genes while KEAP 1 is degraded [82]. Iron metabolism-associated genes (*TFR1*, *FPN*, ferritin heavy chain 1 (*FTH1*), and ferritin light chain (*FTL*)) and heme metabolism-associated genes (*HO-1*, ATP-binding cassette subfamily B member 6 (*ABCB6*), and solute carrier family member 48 member A1 (*SLC48A1*)) are encoded by *Nrf2* target genes in ferroptosis. HO-1 catalyzes heme into ferrous iron and biliverdin; ABCB6 is a key component of heme synthesis; and SLC48A1 acts as a heme transporter. Thus, the activation of Nrf2 can restrict iron uptake, increase iron storage, alleviate electrophilic, and prevent oxidative stress and ferroptosis [8, 80]. Moreover, GSH catalysis and modulation-dependent subunits (*GCLC/GCLM*, glutathione synthetase (*GSS*), and *SLC7A11*) also are Nrf2 transcriptional targets. Nrf2 can motivate the expression of *GPX4* accordingly inhibiting ferroptosis in some cases [29, 83]. Paradoxically, in HCC cell lines, ferroptosis inducers promote *Nrf2* expression, while Nrf2 is a negative regulator of ferroptosis [30].

#### 2.3.5. The Mevalonate Pathway

Several researches find that the mevalonate (MVA) pathway as well as the FSP1/CoQ10/NAD(P)H pathway can serve as independent parallel mechanisms, which synergizing with GPX4 to inhibit lipid peroxidation and ferroptosis [84, 85]. Isopentenyl pyrophosphate (IPP) and antioxidant CoQ10 are two vital products of the MVA pathway. IPP can modulate the synthesis of GPX4 by stabilizing the maturation of selenocysteine tRNA, a necessary regulator for the efficient translation of GPX4 [86]. CoQ10 can be reduced by ferroptosis suppressor protein 1 (FSP1), an oxidoreductase, which also performs as a lipophilic radical-trapping antioxidant that inhibits lipid peroxide propagation. CoQ10 also can be regenerated by FSP1 at the expense of NAD(P)H. The cells with FSP1 knockout are more susceptible to ferroptotic inducers, and this ferroptotic process can be rescued by FSP1 overexpression [85]. Moreover, FSP1 is protective against GPX4 deletion-induced ferroptosis [87]. The investigation of FSP1 further details the pathway of ferroptosis.

#### 2.3.6. The Sulfur-Transfer Pathway

Methionine, a sulfur-containing amino acid, can participate in GSH synthesization after being catalyzed into S-adenosyl homocysteine and cysteine via the sulfur-transfer pathway. Under the situation of cysteine shortage, homocysteine can be transformed into cystathionine (a precursor of cysteine) and then is converted into cysteine to replenish the cysteine pool, getting ready for GSH synthesization [65]. With enough GSH generation, cells can be protected from oxidative injury. The upregulation of sulfur-transfer pathway-related genes can alleviate erastin-induced ferroptosis [88].

#### 2.3.7. NCOA4

Recent studies have reported that ferritin can be degraded in a selective autophagy manner defined as ferritinophagy [39]. NCOA4, a selective cargo receptor, delivers ferritin to lysosomes; then ferritin releases labile iron and increases oxygen radical formation to promote ferroptosis [9, 39]. Classic ATG proteins (ATG3, ATG 5, and ATG7) also are involved in the regulation of ferritin degradation by autophagosome. The knockdown of ATG5, ATG7, or NCOA4 can alleviate erastin-induced ferroptosis by limiting ferritin degradation, iron accumulation, and lipid peroxidation [9].

#### 2.3.8. HO-1

HO-1, an enzyme catalyzing heme into ferrous iron and biliverdin, plays a dual role in ferroptosis regulation. It is reported that HO-1 is cytoprotective which can act as anti-inflammatory, anticancer, antiproliferative, antiapoptotic, and antioxidant media [5]. The overexpression of HO-1 mitigates ferroptosis in the kidney cells and the knockout of HO-1 enhances erastin-induced ferroptosis in these cells [89]. However, the overactivation of HO-1 also can induce ferroptosis by increasing intracellular iron overaccumulation. Under enhanced prooxidant conditions, numerous ferrous iron originating from heme can promote ROS generation, increase lipid peroxidation, and finally induce ferroptosis [43]. The overexpression of HO-1 aggravates erastin-induced ferroptosis in HT-1080 fibrosarcoma cells by offering ferrous iron [90]. Therefore, HO-1 plays a complicated role in the regulation of ferroptosis.

### 2.4. Small-Molecule Modulators of Ferroptosis

Great progresses have been achieved in the present decade to better understand the countless molecules participating in ferroptosis and to provide great opportunities in regulating ferroptosis both chemically and genetically. The following part summarizes various inducers and inhibitors in ferroptotic process. (Table 1).

#### 2.4.1. Inducer

The inducers of ferroptosis can be classified into four categories (e.g., erastin, RSL3, FIN56, and FINO2) according to their mechanisms. (1) System Xc-, a Na^+^-independent cystine/glutamate antiporter, is important to the induction of ferroptosis by controlling the cystine uptake and glutamic acid export [1]. As the prototype ferroptosis inducer, erastin can cause cysteine deprivation and GSH depletion by directly inhibiting system Xc- [1]. Moreover, erastin also can target to the voltage-dependent anion channels (VDACs), resulting in mitochondrial dysfunction and promoting the release of oxides, eventually leading to iron-dependent cell death [21]. (2) GPX4 is a common glutathione peroxidase (GPX) that inhibits the formation of lipid peroxides in ferroptosis. RSL3 can directly inactivate GPX4 by alkylating its selenocysteine, thus inducing lipid peroxidation and ferroptosis [5, 23]. What is more, several studies show that RSL3 and erastin also play a role in the GPX4 protein degradation [91–93]. Withaferin A is a natural inducer for ferroptosis which can inactivate/deplete GPX4 and inactivate Keap1 in neuroblastoma [94]. (3) FIN56 that has a structure of oxime moiety can induce ferroptosis in two different pathways: facilitating the GPX4 degradation and promoting the CoQ10 depletion. The acetyl-CoA carboxylase (ACC) and the enzyme squalene synthase (SQS) participate in FIN56-induced ferroptosis [92]. (4) Ferroptosis induced by FINO2, an organic peroxide, is a comprehensive result of the direct oxidation of labile iron, indirect inactivation of GPX4, and lipid oxidation [95, 96].

#### 2.4.2. Inhibitor

The majority of inhibitors of ferroptosis act through depleting excessive intracellular iron and limiting the formation of lipid peroxides. One category includes deferoxamine (DFO), ciclopirox (CPX), and 2,2′-pyridine, which inhibit ferroptosis by alleviating the accumulation of iron [97]. The second category can block lipid peroxidation by directly deterring the radical chain propagation reaction or indirectly preventing autoinitiation as well as reducing the supply of autoxidizable phospholipids [98]. Thus, maintaining the delicate balance between lipid peroxidation and antioxidant systems is important to regulate ferroptosis. As lipid autoxidation inhibitors, radical-trapping antioxidants (RTAs) can block the propagation of the radical chain reaction directly [99]. Phenols (e.g., butylated hydroxytoluene (BHT)) and aromatic amines (e.g., alkylated diphenylamines), which quickly undergo fast hydrogen atom transfer reactions with peroxyl radicals which are common RTAs [99]. Curcumin and (-)-epigallocatechin-3-gallate (EGCG) can inhibit ferroptosis by alleviating iron accumulation, GPX4 inactivation, GSH depletion, and lipid peroxidation [100]. Vitamin E, as a natural RTA, inhibits ferroptosis in a moderately potent manner [101], while ferrostatin-1 (Fer-1) and liproxstatin-1 (Lip-1) (compounds with aromatic amines) are more potent inhibitors for ferroptosis [4]. Moreover, antioxidants can alleviate ferroptosis via the following mechanisms: (1) the inhibition of Fenton reactions [1], (2) the rescue of oxidative damage [5], (3) the degradation of free radicals [1], and (4) adaptive adjustments [30]. Other categories, such as ferroptosis-related protein synthesis suppressor, *β*-mercaptoethanol (a reducing agent), selenium, DPP4 activity inhibitor, and ebselen (GPX suppressor), can also inhibit the ferroptotic process [19].

### 2.5. Role of Ferroptosis in Pulmonary Disease

Emerging evidences elucidate that ferroptosis is a key process for various disease including cancer (e.g., lung, breast, and lymphoma), degenerative diseases (e.g., Parkinson's, Alzheimer's, and Huntington's diseases), metabolic disease, ischemia-reperfusion injury, stroke, intracerebral hemorrhage, and renal failure [19]. The involvement and application of ferroptosis in pulmonary diseases also receive much consideration. The modulation of ferroptosis (positive or negative) is of great attention for the treatment of ferroptosis-associated disease: the induction and suppression of ferroptosis can be an efficient therapy for refractory tumors and degenerative disease, respectively [95, 102]. Thus, based on the mechanism of ferroptosis mentioned above (Figure 1), this review summarizes the role of ferroptosis in several specific pulmonary diseases (Table 2).

#### 2.5.1. Pulmonary Infection

Continuous studies find that ferroptosis plays a role in bacterial infection-associated lung injury. One study reals that lipoxygenase (pLoxA) secreted by *Pseudomonas aeruginosa*, a prokaryotic bacterium, can oxidize PUFA-PE, induces lipid peroxidation and initiates ferroptosis in host bronchial epithelium cells. Moreover, the ferroptosis induced by clinical *P. aeruginosa* isolates is pLoxA dependent [103]. Another study demonstrates that *Mycobacterium tuberculosis*- (Mtb-) infected macrophage also has several ferroptotic characteristics including increased free iron, glutathione and GPX4 reduction, mitochondrial superoxide, and lipid peroxidation accumulation. Besides being an antioxidant inhibitor, Fer-1 can obviously alleviate Mtb-induced macrophage and mouse pulmonary necrosis which is correlated with limited GPX4 expression and enhanced lipid peroxidation. More importantly, Fer-1 can also reduce the bacterial burden of infected animals, providing new therapeutic opportunities for tuberculosis [104].

#### 2.5.2. COPD

Globally, chronic obstructive pulmonary disease (COPD), the fourth leading cause of morbidity and mortality, remains unclear and has no curative treatment [105]. Chronic cigarette smoke (CS) is a high risk factor for the initiation of COPD. A previous study shows that cigarette exposure increases iron concentrations in lavage and elevates the level of ferritin both in lavage and in serum from the lung of rats and COPD. The deposition of iron and other particulate matter in smokers' lung can intervene with oxidative stress by regulating iron homeostasis [106]. Whole cigarette smoke condensates (WCSC) can induce ferroptosis in bronchial epithelial cells [107]. Another study reveals that CS facilitates ferroptosis by inducing NCOA4-mediated ferritinophagy in lung epithelial cells. While Gpx4 knockdown dramatically aggravates CS-induced COPD, reducing iron intake or using iron chelators remarkably alleviates CS-induced COPD [108].

#### 2.5.3. Pulmonary Cancer

Iron dysregulation is gradually deemed as a risk factor for lung cancer [109, 110]. It is reported that the expression of ferritin and TFR1 is elevated at a percentage of 62% and 88% among the patients with non-small-cell lung cancer (NSCLC). The NSCLC and small cell lung cancer (SCLC) patients also have elevated serum ferritin [111, 112]. The expression of FSP1 can promote ferroptosis resistance in lung cancer cells from mouse tumor xenografts or culture [85]. Under the circumstance of intracellular ROS accumulation, the suppression of NFS1 can promote ferroptosis in lung adenocarcinomas, while the inhibition of NFS1 alone (without excessive ROS) has little effect on ferroptosis [17]. One study has revealed that erastin can promote the expression of *p21* and *Bax* by enhancing and activating p53, subsequently inhibits SLC7A11 activity, accelerates ROS accumulation, and eventually induces ferroptosis as well as apoptosis in A549 [113]. Other studies illustrate that Nrf2 is a major regulator for the induction of NSCLC cell ferroptosis even N5CP (NSCLC cells resistant to CDDP) cell ferroptosis. Acetaminophen (APAP) along with erastin can induce NSCLC cell ferroptosis and apoptosis by targeting Nrf2 [114]. Moreover, CDDP along with low doses of erastin or sorafenib can effectively induce ferroptosis in N5CP cell [115]. These studies reveal that crosstalks between ferroptosis and other RCDs (e.g., apoptosis) do exist in pulmonary cancer. Some agents lead to cell death by working on different cell death pathways, and targeting ferroptosis can circumvent the limitations of some traditional anticancer therapies experimentally. Therefore, a comprehensive investigation of ferroptosis and its correlations with other cell death pathways would provide a novel treatment therapy for patients with pulmonary cancer, even drug-resistant cancer.

#### 2.5.4. Pulmonary Fibrosis

Disruption of the redox balance is deemed as a key factor in the initiation and development of many airway pathologies. So far, the mechanism of pulmonary fibrosis is not yet fully understood. Increased ROS accumulation and GSH depletion, which are tightly associated with ferroptotic process, also play critical roles in the pathogenesis of pulmonary fibrosis. Ferroptosis is found to participate in acute radiation-induced lung fibrosis (RILF), and the ROS accumulation seems to be the primary inducer of ferroptosis in this process [116, 117]. The administration of Lip-1 can alleviate pulmonary fibrosis by decreasing ROS, limiting collagen deposition, and reducing HYP. The decreased GPX4 induced by irradiation in RILF can be mitigated by Lip-1 as well. Under the treatment of the ferroptosis inhibitor, the level of transforming growth factor-*β*1 (TGF-*β*1), a key profibrogenic factor, also decreased significantly in acute RILI. Moreover, erastin can promote fibroblast-to-myofibroblast differentiation by inhibiting the GPX4 expression and accelerating lipid peroxidation in HFL1 cell line [118]. Other studies suggest that ferroptosis is involved in paraquat- (PQ-) induced pulmonary damage and ferroptosis inhibitors might be promising therapeutics in alleviating PQ poisoning [119]. It is also found that ferroptosis is involved in the fibrosis of other organs including the liver and heart [120, 121].

#### 2.5.5. Asthma

Recent studies confirm the involvement of T2 inflammation with IL-4/IL-13-driven accelerated 15-lipoxygenase 1 (15-LO1) expression and PEBP1 binding in vivo to the exacerbation of asthma [122]. Although the 15-LO1 is upregulated and highly colocalized with PEBP1 in human airway epithelial cells (HAECs) from stable, nonexacerbating patients, the oxygenated PEs can be degraded by GPX4. Inactivation of GPX4 by RSL3 in IL-13-cultured HAECs induced the accumulation of abundant oxygenated PEs. Moreover, the blockage of PEBP1 in HAECs can decrease ferroptotic sensitivity. Thus, enhancing GPX4 at the initiation of asthma exacerbation might prevent ferroptosis and alleviate asthma [16].

## 3. Conclusions

As a unique RCD modulating human health, ferroptosis brings some challenges, which are also accompanied with great opportunities. This review summarizes the mechanisms of ferroptosis (Figure 1) and discusses its role in various pulmonary disease processes, including infection, COPD, cancer, fibrosis, and asthma (Table 2). Although increasing researches have confirmed that ferroptosis plays an intricate role in human health, the role of ferroptosis in pulmonary disease remains in its relative infancy.

The main mechanisms of ferroptosis rely on intracellular iron accumulation, GSH depletion, GPX4 inactivity, and excessive lipid hydroperoxide production [1]. In addition to the key ferroptotic modulating pathways, multiple small molecules (erastin, RSL3, FIN56, FINO2, iron chelation, and RATs) are also involved in the regulation of ferroptosis [1]. Recent pulmonary researches coincide with these findings, which confirm that respiratory disease could be modulated by ferroptotic inducers (erastin, RSL3, and Zn) or inhibitors (Fer-1, DFO, and Lip-1) [103, 104, 108, 114–118]. Moreover, some combined therapies targeting the underlying crosstalks between ferroptosis and other cell death phenotypes provide a greater value in pulmonary disease experimentally. However, the majority researches on ferroptosis-related pulmonary diseases based on animal models and the detailed effects of ferroptotic therapy in patients with pulmonary disease remain greatly unknown. The gene expression and the sensitivity of ferroptosis in different cell lines may differ greatly. Whether these signaling pathways are common on ferroptosis-related pulmonary diseases or cell/species/disease-specific has not yet been fully investigated. Thus, despite the fast growth of achievements on ferroptosis in animal models with pulmonary disease, some challenges remain to be overcome in its clinical applications, which are also accompanied with great potentials.

It is believed that ferroptosis has a great theoretical significance and a practical value in the occurrence, development, and treatment of human diseases and further exploration will open up a new platform in designing ferroptosis-based therapeutic intervention. The benefits of ferroptosis upon pulmonary disease deserves priority in a future research.

## Figures and Tables

**Table 1:** The common inducers and inhibitors of ferroptosis.

	Mechanisms	Drugs or compounds	Reference
Inducer	Inhibit system Xc- and prevent cystine import	Erastin and derivatives, SAS, sorafenib	[1, 22, 68]
	Inhibit GPX4	RSL3	[5, 23]
	Degrade GPX4, bind to SQS, and deplete antioxidant CoQ10	FIN56	[92]
	Oxidize ferrous iron and lipidome directly, inactivate GPX4 indirectly	FINO2	[96]
	Target VDACs, degrade GPX4	Erastin	[21, 91]
	Inactivate/deplete GPX4, inactivate Keap1	Withaferin A	[94]
	Inhibit cystine uptake	Glutamate	[22]
	GSH depletion	Buthionine sulfoximine, cisplatin	[5, 66]
Inhibitor	Inhibit accumulation of iron	DFO, CPX, 2,2′-pyridine	[97]
	Catalytic RTA, prevention of lipid peroxidation	Fer-1, Lip-1, nitroxide-based compounds	[4]
	Lipophilic antioxidant compensating GPX4 loss	Vitamin E	[101]
	Prevent iron accumulation, GPX4 inactivation, GSH depletion, and lipid peroxidation	Curcumin, EGCG	[100]
	Prevent GSH depletion and lipid peroxidation	Baicalein, NDGA	[62, 63]

CoQ10: coenzyme Q10; system Xc-: cysteine/glutamate transporter receptor; CPX: ciclopirox; DFO: deferoxamine; EGCG: (-)-epigallocatechin-3-gallate; Fer-1: ferrostatin 1; FIN 56: ferroptosis inducing agent 56; GSH: glutathione; GPX4: glutathione peroxidase 4; Keap1: Kelch-like ECH-associated protein 1; Lip-1: liproxstatin-1; NDGA: nordihydroguaiaretic acid; RTA: radical-trapping antioxidant; RSL3: RAS-selective lethal 3; SQS: squalene synthase; SAS: sulfasalazine; VDACs: voltage-dependent anion channels.

**Table 2:** The role of ferroptosis in lung disease pathogenesis.

Disease	In vitro model	In vivo model	Mode of action	Pathogenesis	Regulator	Reference
Infection						
P. aeruginosa infection	HBECs, bacterial strains. P. aeruginosa WT	P. aeruginosa ICU respiratory isolates	Express pLoxA, oxidize membrane PLs (especially AA-PE), produce 15-HOO-AA-PE	Biofilm formation, colonization	Inducer: RSL3	[103]
Tuberculosis	Murine BMDMs, human monocyte-derived macrophages	Mice	Reduce glutathione and GPX4 Increase free iron, mitochondrial superoxide, and lipid peroxidation	Pulmonary necrosis, bacterial load	Inhibitors: Fer-1, iron chelation	[104]
COPD						
	HBECs, BEAS-2B, A549	Mice	Promote NCOA4-mediated ferritinophagy, ER stress, mitochondrial dysfunction	Increase of DAMPs, ROS lipid peroxidation, emphysema	Inhibitors: DFO, Fer-1, Lip-1	[107, 108]
Cancer	HBECs, NCI-H1299, A549, H460, SPC-A-1, PC9, SW900, SK-LU-1, WI-38, H358, Calu-1	NA	Promote p53 Reduce GPX4, inhibit Nrf2/Xc-, FSP1	Inhibition of tumorigenesis	Inducers: erastin, RSL3, Zn Inhibitor: DFO	[17, 85, 113–115]
Fibrosis						
RILF	NA	Mice	Reduce GPX4, Nrf2, HO-1 and NQO1 Promote ROS lipid peroxidation, TGF-*β*1 and FMT	Inflammasome activation, ECM deposition, fibrosis	Inhibitor: Lip-1	[116, 117]
	HFL1	NA	Inhibit GPX4 Promote lipid peroxidation, TGF-*β*1 and FMT	ECM deposition, FMT	Inhibitor: Lip-1 Inducer: erastin	[118]
PQ poisoning	NA	NA	Promote ROS, lipid peroxidation, NF-*κ*B activation, mitochondrial damage	Redox imbalance, structural failure, lung fibrosis	NA	[119]
Asthma	HAECs, MLE-12	Rats	Oxidize PE by PEBP1	Th2 inflammation	Inducer: RSL3 Inhibitor: Fer-1	[16, 122]

AA-PE: acid phosphatidylethanolamines; BMDMs: bone marrow-derived macrophages; COPD: chronic obstructive pulmonary disease; system Xc-: cysteine/glutamate transporter receptor; DAMP: damage-associated molecular patterns; DFO: deferoxamine; ECM: extracellular matrix; Fer-1: ferrostatin-1; FSP1: ferroptosis suppressor protein 1; GPX4: glutathione peroxidase 4; HO-1: hemeoxygenase-1; ICU: intensive care unit; pLoxA: lipoxygenase; Lip-1: liproxstatin-1; Nrf2: nuclear factor erythroid-derived 2; NF-*κ*B: nuclear factor kappa beta; NCOA4: nuclear receptor coactivator 4; PQ: paraquat; PEBP1: phosphatidylethanolamine-binding protein 1; PE: polyunsaturated phosphatidylethanolamines; NQO1: quinone oxidoreductase 1; RILF: radiation-induced lung fibrosis; RSL3: RAS-selective lethal 3; ROS: reactive oxygen species; Th2: T helper type 2; TGF-*β*1: transforming growth factor-*β*1; WT: wild type; Zn: zinc; 15-HOO-AA-PE: 15-hydroperoxy-AA-PE.
